# Adrenocorticotropin-Dependent Ectopic Cushing's Syndrome: A Case Report

**DOI:** 10.7759/cureus.49124

**Published:** 2023-11-20

**Authors:** André Rebelo Matos, André Martins, Maria J Barbosa, Inês Quinteiro, Diogo Faustino, Rita Gameiro, Luísa Azevedo

**Affiliations:** 1 Internal Medicine Department, Hospital São José, Centro Hospitalar Universitário de Lisboa Central, Entidades Públicas Empresariais (EPE), Lisbon, PRT; 2 Critical Care Medicine Department, Hospital São José, Centro Hospitalar Universitário de Lisboa Central, Entidades Públicas Empresariais (EPE), Lisbon, PRT

**Keywords:** small-cell lung cancer, hypokalemia, ectopic, cushing’s syndrome, paraneoplastic syndromes

## Abstract

Paraneoplastic syndromes are rare and diverse conditions caused by either an abnormal chemical signaling molecule produced by tumor cells or a body’s immune response against the tumor itself. These syndromes can manifest in a variable, multisystemic and often nonspecific manner posing a diagnostic challenge.

We report the case of an 81-year-old woman who exhibited severe hypokalemia, metabolic alkalosis, and worsening hyperglycemia. The investigation was consistent with adrenocorticotropin (ACTH)-dependent Cushing’s syndrome and, eventually, the patient was diagnosed with stage IV primary small-cell lung cancer (SCLC).

SCLC is known to be associated with paraneoplastic syndromes, including Cushing’s syndrome caused by ectopic adrenocorticotropin (ACTH) secretion. Despite being associated with very poor outcomes, managing these syndromes can be challenging and may hold prognostic significance.

## Introduction

Adrenocorticotropin (ACTH)-dependent Cushing’s syndrome (CS) is caused by excessive ACTH production by corticotroph (Cushing’s disease (CD)) or nonpituitary (ectopic) tumors, leading to excessive cortisol production. Ectopic ACTH syndrome (EAS) is a rare condition, accounting for 10 to 20% of all cases of ACTH-dependent CS and 5 to 10% of all types of CS [1]. The normal glucocorticoid-induced suppression of ACTH is reduced in ACTH-dependent CS, especially with ectopic ACTH production. Studies show that a wide variety of neoplasms, usually carcinomas rather than sarcomas or lymphomas, have been associated with EAS. Most cases are caused by neuroendocrine tumors of the lung, pancreas, or thymus, in which the hypercortisolism state is not apparent clinically, resulting, all too often, in delayed diagnosis [2,3].

Current diagnostic tests for EAS aim to confirm high cortisol levels, the absence of a cortisol circadian rhythm, as well as the reduced response to negative feedback from glucocorticoid administration, and imaging to identify the site of ACTH production.

Prompt diagnosis and management are crucial in EAS, highlighting the importance of physician awareness and early recognition of this syndrome.

Treatment options depend on the underlying tumor. Surgical removal is often the primary approach, followed by radiation therapy or chemotherapy. Additionally, medications to control cortisol levels may be necessary to manage the various comorbid conditions associated with CS, such as cardiovascular disease, diabetes, electrolyte imbalances, infections and thrombotic risk [4,5].

## Case presentation

We report the case of an 81-year-old woman with a fully active performance status (ECOG 0) and a medical history of diabetes, hypertension, dyslipidemia, and depressive disorder. She was admitted to an internal medicine ward due to an acute hydroelectrolytic disorder, including metabolic alkalosis, severe hypokalemia (2 mmol/L), hypochloremia (85 mmol/L), hypocalcemia (0.95 mmol/L), hypophosphatemia (1.4 mg/dL), hypomagnesemia (0.9 mg/dL), and hyperlactatemia (5.8 mmol/L), after she reportedly self-medicated herself with higher doses of metformin (four to five pills a day) due to high blood glucose levels. The patient presented with asthenia, nausea, vomiting, and diarrhea for three days and reported uncontrolled blood glucose levels for the last eight days. 

The physical examination was unremarkable, without any altered mental status or signs of infection. Arterial blood gas samples showed metabolic alkalemia (pH 7.59) and hyperlactatemia, associated with severe hypokalemia, normal bicarbonate (27 mmol/L), and mildly elevated glycemia and ketonemia (232 mg/dL and 1.7 mmol/L, respectively). Lab tests confirmed the serum potassium levels as well as the other aforementioned electrolyte disturbances. Kidney function and hepatic enzymes were normal. Considering the possible relationship between the electrolyte disorder and the gastrointestinal presentation, the patient was given intravenous (IV) fluids and received potassium and magnesium replacement therapy.

**Figure 1:**
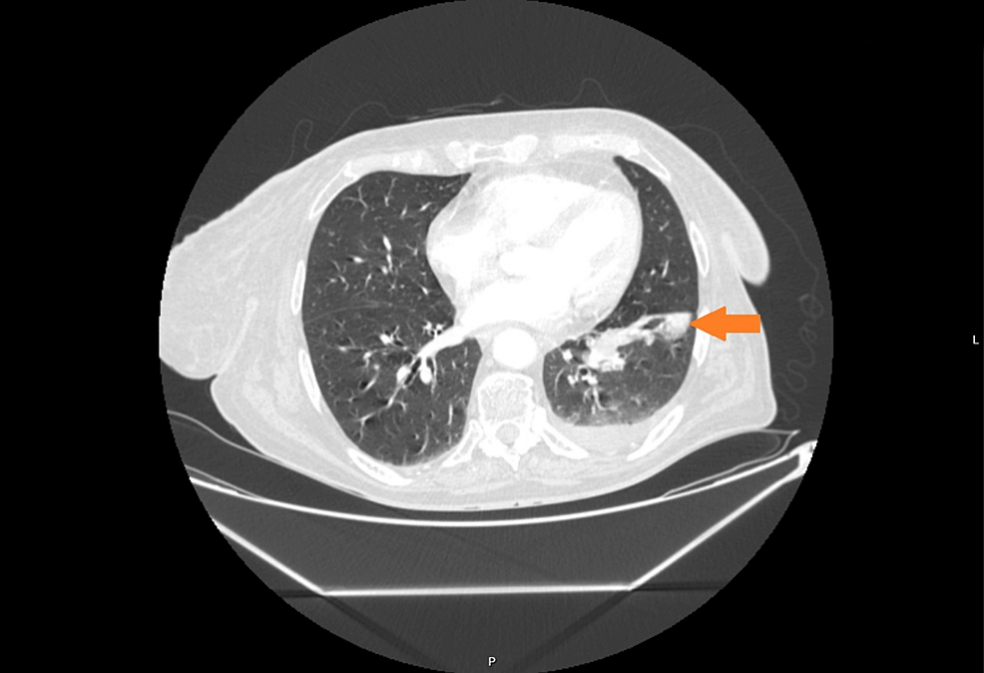
Pulmonary mass (SCLC) in the left lower lobe with ipsilateral hilar lymphadenopathy and pleural effusion. SCLC: small-cell lung cancer.

**Figure 2:**
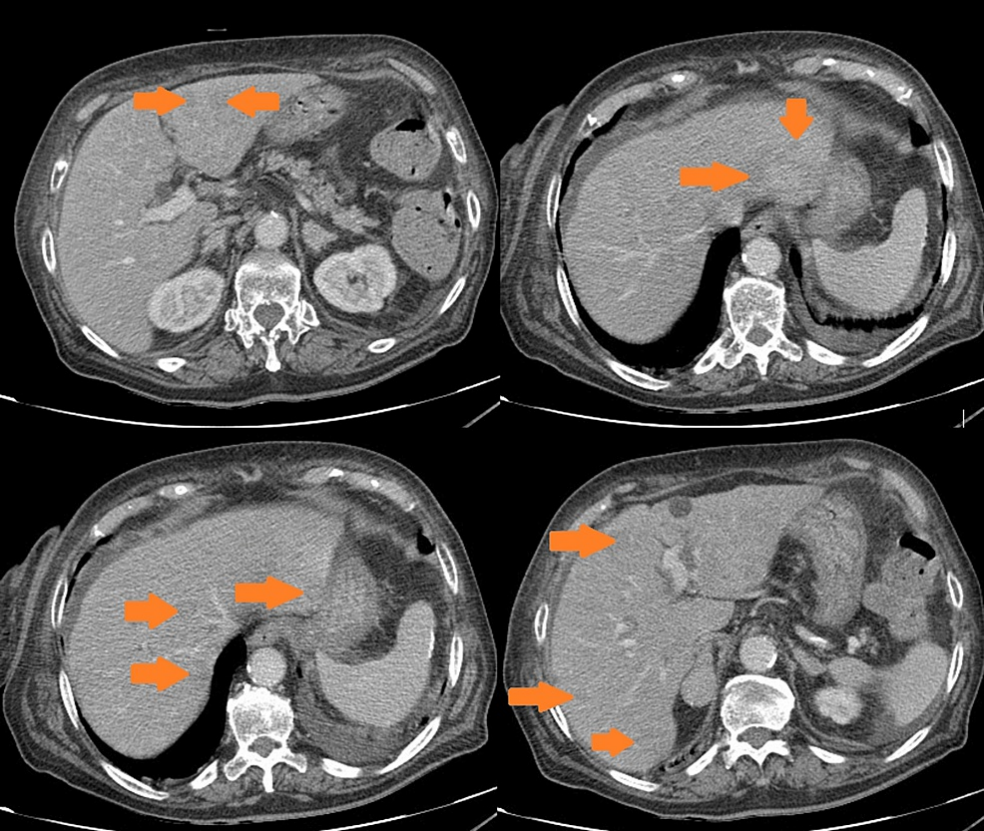
Secondary involvement of the liver with hypodense multilobar hepatic lesions (arterial phase).

**Figure 3:**
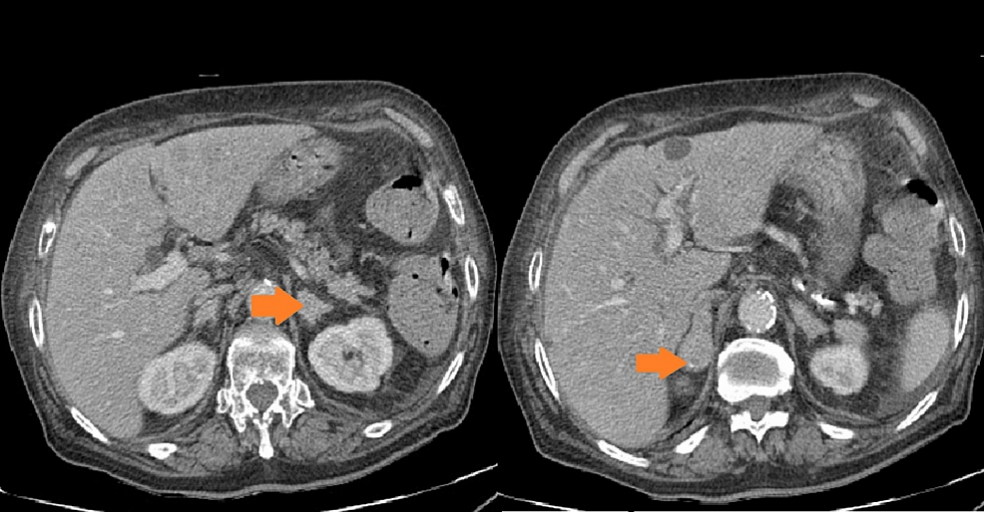
Bilateral suprarenal lesions suggestive of secondary involvement.

Despite receiving 200 milliequivalents (mEq) of IV potassium chloride and 4 grams of magnesium sulfate, in the first 48 hours, the ion deficits persisted. Given the persistent electrolyte derangement, the patient was admitted to the Internal Medicine ward for etiological investigation and monitoring of ionic correction. The initial period was remarkable for refractory hypokalemia and uncontrolled diabetes under respective therapeutic measures, including 80 to 130 mEq of IV potassium chloride and progressive titration of spironolactone to 200 mg a day. Laboratory investigation revealed high parathormone levels (PTHi 167 pg/mL; reference range: 10-65 pg/mL), vitamin D deficiency (3.3 ng/mL; reference range >20 ng/mL) and apparent ACTH-dependent hypercortisolism (serum cortisol 80.20 ug/dL; ACTH 445 pg/mL), as well as high urinary potassium and glucose concentrations (190 mEq/24 h and 21161 mg/24 h). A dexamethasone suppression test was performed twice (standard low and high dose) without any changes in cortisol levels, leading to the suspicion of a CS caused by abnormally high ACTH production. Cranioencephalic computed tomography (CT) and magnetic resonance imaging (MRI) were performed, excluding the presence of pituitary anomalies. A follow-up whole-body CT scan was performed, revealing a suspicious pulmonary mass in the left lower lobe, associated with ipsilateral hilar lymphadenopathy and hepatic and adrenal gland lesions suggestive of secondary involvement. An endobronchial ultrasound bronchoscopy and biopsy were performed, documenting anatomopathological findings of small-cell lung carcinoma with a Ki67 expression of 100% (Figures 1-3).

The patient was referred to oncology, and chemotherapy was deferred, considering the infectious risk associated with hypercortisolism.

The patient started metyrapone 500 mg every eight hours, resulting in a reduction in cortisol levels and control of hypokalemia. Later on, a fluorodeoxyglucose-positron emission tomography (FDG-PET) scan was performed, confirming disseminated disease with additional bone involvement. Unfortunately, despite endocrinological stabilization, the patient's condition worsened, and she ended up dying one month after the diagnosis. 

## Discussion

When this patient was admitted, it was assumed that the metabolic alkalosis and various electrolyte disturbances were related to the gastrointestinal presentation and hyperlactatemia secondary to metformin overdose. However, the unusual persistence and refractory hypokalaemia raised some concerns that an alternative etiology might be involved and incited subsequent testing.

The high cortisol levels were unexpected given the subclinical presentation, which seems to be more frequent in cases of EAS. In fact, because of this, the true incidence of EAS is unknown and probably underdiagnosed since patients often have subclinical presentations and do not exhibit catabolic features.

Since the patient wasn’t on any steroid medication, the association between the high cortisol and ACTH levels, non-responsive to the dexamethasone suppression test, along with the absence of a pituitary lesion, raised suspicion of a probable EAS, which was later confirmed by the body CT scan and endobronchial ultrasound (EBUS).

EAS is a rare disease with a poor prognosis. It reportedly occurs in 3.2 to 6% of neuroendocrine neoplasms, and the tumor often originates in the lung, thyroid, stomach, and pancreas. Locoregional and/or distant metastasis can be seen at the time of diagnosis in 15% of typical carcinoids and about half of atypical carcinoids with visible primaries [6,7].

The presence of a typical CS presentation, with or without electrolyte abnormalities, should raise suspicion and serum levels of both ACTH and cortisol should be assessed to determine if they are elevated and to distinguish between an ACTH-dependent (pituitary or nonpituitary ACTH-secreting tumor) and an independent mechanism (e.g., from an adrenal source). The diagnosis of CS is established when at least two different first-line tests are unequivocally abnormal and cannot be explained by any other conditions that cause physiologic hypercortisolism. Additional evaluation is performed to rule out a pituitary origin (with brain MRI) and to assess for a possible ectopic ACTH-secreting tumor.

In the aforementioned case, the production of ACTH was caused by primary neuroendocrine SCLC. The recommended approach to EAS involves the initial normalization of serum cortisol levels and the treatment of related comorbidities before performing a complete diagnostic evaluation and addressing the underlying cause [5-7]. This approach seems to improve survival and prevent complications such as sepsis following a combined steroid-induced immunosuppression and chemotherapy-induced agranulocytosis [6,7].

Direct therapies vary according to the tumor, but surgery is usually the first line of treatment (transsphenoidal surgery in cases of CD or tumor resection in cases of non-metastatic EAS). However, our patient presented with stage IV SCLC with EAS, in which chemotherapy remains the first-line treatment. SCLC patients with EAS have a poorer prognosis than those without EAS, with a life expectancy of only three to six months. This makes early diagnosis more important [2,7], as controlling the high cortisol levels and then administering systemic chemotherapy may achieve longer survival [8].

Apart from systemic chemotherapy, ketoconazole (widely accepted but highly toxic), metyrapone, mitotane (adrenocortical suppressant drug with significant side effects), and mifepristone (glucocorticoid antagonist, mainly used for the treatment of hyperglycemia in CS) can be used to reduce circulating glucocorticoids. Moreover, thromboprophylaxis and *Pneumocystis jirovecii* pneumonia prophylaxis should be started.

Because ketoconazole may increase the risk of chemotherapy toxicity by inhibiting cytochrome P450 3A4, metyrapone has been reported to be a better choice [5,7].

Nonetheless, administration of chemotherapy in the setting of a hypercortisolism-induced immunosuppressive state, cancerous background and metabolic disorders featuring electrolyte disturbance and hyperglycemia, aggravate the condition and can be life-threatening. Thus, a palliative approach can sometimes be reasonable.

## Conclusions

The diagnosis of CS is a three-step process that includes its suspicion based on the patient's laboratory and semiologic findings, the documentation of hypercortisolism, and the identification of its cause, which can be either ACTH-dependent or independent. 

The ectopic secretion of ACTH (EAS) by nonpituitary tumors is a relatively rare cause of CS and often presents as paraneoplastic syndromes, adding therapeutic and prognostic concerns.

This case, in particular, highlights the importance of seeking alternative explanations for common electrolyte disturbances, particularly when they don't resolve promptly. Clinicians should be aware of EAS and its frequent subclinical presentation in order to initiate the diagnostic workup as soon as suspicion arises.
